# Genome-Wide Analysis of DoSPX Genes and the Function of *DoSPX4* in Low Phosphorus Response in *Dendrobium officinale*

**DOI:** 10.3389/fpls.2022.943788

**Published:** 2022-07-11

**Authors:** Lin Liu, Haoxin Xiang, Jingjing Song, Huimin Shen, Xu Sun, Lingfeng Tian, Honghong Fan

**Affiliations:** School of Life Sciences, Anhui Agricultural University, Hefei, China

**Keywords:** gene family, SPX, PHR, *Dendrobium officinale*, gene function

## Abstract

*Dendrobium officinale* Kimura et Migo is a famous Chinese herb. *D. officinale* grows on rocks where the available phosphorus is low. The SPX family plays a critical role in maintaining Pi homeostasis in plants. In this paper, 9 SPX family genes were identified in the genome of *D. officinale*. Bioinformatics and qRT-PCR analysis showed that DoSPXs were involved in response to −Pi stress and had different expression patterns. *DoSPX4*, which had a unique expression pattern, was clustered with AtSPX4 and OsSPX4. Under −Pi treatment, the expression level of *DoSPX4* reached a peak on 5 d in roots, while showing a downward trend in the aboveground parts. DoSPX4 was located on the cell membrane. Overexpression *DoSPX4* promoted Pi content in the stem and the expression level of *NtPHT1/2* in *Nicotiana tabacum*. The results of Yeast two-hybrid showed that DoSPX4 could interact with Phosphate High-Affinity Response factor (DoPHR2). These results highlight the role of *DoSPX4* in response to low phosphorus, which provides a theoretical basis for further study on the response mechanism of −Pi in *D. officinale*.

## Introduction

Phosphorus (Pi) plays an essential role in plant growth (Chen and Liao, 2017). The low content of available Pi in the soil is difficult to meet the Pi requirements accompany with plant growth. Plants increase effective Pi utilization by sensing and absorbing Pi in the soil, then adapt to low Pi environment in molecular and physiological levels.

*Dendrobium officinale* is a perennial herb which has a variety of pharmacological effects. It can grow on tree trunks, rocky cliffs, or fern surfaces in natural conditions. It is subjected to various environmental stresses in the harsh growth environment, among which low Pi stress is the main factor affecting its growth and development (Liu N. et al., 2018). The previous research found that *D. officinale* could respond to −Pi and accumulate effective active components in low phosphorus. The expression of the key genes in the secondary metabolism were significantly correlated with Pi concentration, while the early response genes to −Pi in *D. officinale* have not been reported (Liu L. et al., 2021).

Proteins, containing the SPX domain, have been identified as early response factors which participate in Pi signal transduction in plants (Zhou et al., 2021). The N-terminal of plant SPX protein contains a highly conserved SPX domain, named by the first letter of SYG1, PHO81, and XPR1 genes (Secco et al., 2012). At present, SPX has been classified into four subfamilies by the C-terminal domains of the protein. The four subfamilies are SPX (containing only one SPX domain), SPX-EXS (containing SPX and one EXS domain), SPX-MFS (containing SPX and MFS domain), and SPX-RING (containing ring-type star finger domain) (Wang et al., 2004; Chen et al., 2009; Chiou and Lin, 2011; Lin et al., 2013; Su et al., 2015; Wang et al., 2015; Liu J. et al., 2016; Zhang et al., 2016; Yue et al., 2017; Yang et al., 2018). Among them, the SPX subfamily plays an important role in the early stage of Pi signal recognition.

Generally, SPX can respond to Pi signals and then interact with MYB to change the transcriptional activation of downstream *PSI* genes. At present, Phosphorus-related MYB includes PHR (MYB-CC) and several R2R3-MYB. In *Arabidopsis*, *AtSPX*1/2/3/4 proteins are upstream regulators of *AtPHR*1 (Duan et al., 2008). *AtSPX*1 and *AtSPX*2 had functional redundancy. Both of them could regulate *AtPHR*1, and the extent of this interaction was affected by *AtSPX*3 (Puga et al., 2014). In stem, *AtSPX4* was a repressor of *AtPHR1* (Osorio et al., 2019). In *Oryza sativa*, there were 6 SPXs (OsSPX1–OsSPX6). Under Pi deficiency, the expression of *OsSPX4* was downregulated, while the other five *OsSPX* genes showed an upward trend (Secco et al., 2012; Lv et al., 2014). *OsSPX*1/2/4 could interact with *OsPHR*2 and affect the regulation of *OsPHR*2 on downstream *PSI* genes (Shi et al., 2014; Wang et al., 2014). *OsSPX3*, *OsSPX5*, and *OsSPX6* were homologous genes, which were involved in functional redundancy in response to phosphorus. *OsSPX*3 and *OsSPX*5 could form homodimers and participate in complex regulation in *O. sativa* (Shi et al., 2014). OsSPX4 was rapidly degraded by the proteasome pathway under low phosphorus, which had unique subcellular localization (Lv et al., 2014). These studies showed that SPX played an important role in phosphate response in plants, and *SPX*4 (*OsSPX*4 in *O. sativa* and *AtSPX*4 in *A. thaliana*) may have a different regulatory pattern from other SPXs.

Among them, gene prediction based on similarity comparison through published genome sequence has become the main method to screen key genes and analyze molecular mechanisms (Bhatt et al., 2021). It has become the main method to screen key genes and analyze molecular mechanism that gene prediction based on similarity comparison. The previous studies showed that low phosphorus could promote the accumulation of effective active substances in *D. officinale* (Liu L. et al., 2021). However, the molecular mechanism of *D. officinale* response to −Pi has not been reported. In this study, the bioinformatics analysis of the early Pi response factor DoSPX was carried out. The expression pattern of *DoSPX* in *D. officinale* was analyzed by qRT-PCR. DoSPX4 had a unique expression pattern, which was subcellular localization was further investigated. The interaction between DoSPX4 and DoPHR2 was studied by Yeast two-hybrid. The function of *DoSPX4* to plant low phosphorus was verified by heterologous overexpression of *DoSPX4* in *N. tabacum*.

## Materials and Methods

### Treatment and Preservation of Plant Materials

The *D. officinale* tissue culture seedlings used in the experiment were from the Anhui Provincial Engineering Technology Research Center for Development and Utilization of Regional Characteristic Plants, School of Life Sciences, Anhui Agricultural University. The seedlings of *D. officinale* with uniform size, shape, and color were cultured in the plant tissue culture room under a constant temperature of 25°C at 8L:16D photoperiod. The tissue culture seedlings were cultured on Murashige and Skoog medium (MS) for 8 months, then put into MS medium with different Pi concentrations. The 5 levels of KH_2_PO_4_ are used to set different Pi concentrations on MS medium (2.5, 1.25, 0.625, 0.0625, and 0 mM), and K^+^ in different Pi concentration media were supplemented with different concentrations of KCl. Samples were taken on 0, 1, 5, 10, and 40 days after treatment and put into a 10-ml centrifuge tube and immediately froze in liquid nitrogen and store it in the refrigerator at −80°C for standby. The three biological replicates were set for each treatment.

### Identification of SPX Gene Family in *Dendrobium officinale*

By reference SPX protein sequences in *Oryza sativa* (Supplementary Table 1) and *Arabidopsis thaliana* (Supplementary Table 1), SPX proteins in *D. officinale* genome (Supplementary Table 1) sequence were selected with a threshold of *E*-value < 1E–5. Then the obtained sequences were submitted to CD-HIT^1^ (Fu et al., 2012) to remove the redundant sequence. Finally, the candidate sequences are submitted to SMART^2^ (Letunic et al., 2021) and PFAM^3^ (Mistry et al., 2021) to identify the conserved motifs. The basic information of protein sequence was obtained online by the ExPasy website^4^ (Gasteiger et al., 2003).

### Construction of DoSPXs Phylogenetic Tree

The 7 SPX sequences in *D. officinale* were compared with the SPX protein sequences of 27 *A. thaliana*, 12 *O. sativa* and 12 *Phalaenopsis equestris* by ClustalW (Thompson et al., 1994). A phylogenetic tree was constructed using the MEGA7.0 NJ method with the bootstrap = 1,500 and beautified with ITOLS online website^5^ (Letunic and Bork, 2021). The names of the gene name and accession numbers of SPX can be found in Supplementary Table 2.

### *Cis*-Acting Element Analysis

The TBTOOLS (Chen et al., 2020) was used to extract the promoter region of the genome sequence by using the genome annotation file of *D. officinale*. The 1,500 bp upstream sequences of SPX CDS was submitted to PLANTCARE^6^ (Lescot et al., 2002) and NEWPLACE^7^ (Higo et al., 1999) online websites to analyze the possible *cis*-acting elements of the promoter. Use TBTOOLS for drawing.

### Subcellular Localization

In order to understand the subcellular localization of *DoSPX4*, the recombinant plasmid pCAMBIA1305-DoSPX1-GFP was constructed by amplifying the fragment of DoSPX4 by sense and antisense primers (Supplementary Table 3). The recombinant plasmid was introduced into *Agrobacterium tumefaciens* EHA105. EHA105 Infect tobacco leaves and *DoSPX4* is transiently expressed in *Nicotiana benthamiana* leaf epidermal cells. A confocal laser microscope was used to observe the GFP fluorescence signal.

### Yeast Two-Hybrid

In order to verify the interaction of DoSPX4 with DoPHR2, the AD-DoSPX4 recombinant vector was constructed by amplifying DoSPX4 open reading frame into AD, and the DoPHR2-BD recombinant vector was constructed by amplifying the DoPHR2 fast-play reading frame into BD, the primers were shown in Supplementary Table 3. The constructed AD-DoSPX4 and DoPHR2-BD were used to transform AH109 jointly, and the successfully transformed clones were screened on SD-WL, and the clones were screened on SD-HAWL. The X-α-Gal is used to identify positive interactions (SD-WL indicates the SD medium without Leu and Trp, and SD-HAWL means the SD medium without Ade, His, Leu, and Trp).

### *Nicotiana tabacum* Transformation

In order to understand the function of *DoSPX4*, the recombinant plasmid pCAMBIA1305-*DoSPX4* was constructed by amplifying the fragment DoSPX4 by sense and antisense primer (Supplementary Table 3), pCAMBIA1305-*DoSPX4* transformed into *A. tumefaciens* GV3101. The GV3101 was transformed into *N. tabacum* leaf discs *via* an *A. tumefaciens*-mediated leaf disc procedure (Topping, 1988). Regenerated plants are obtained by inducing callus, budding, rooting, and transplanting. The positive strain is screened and selected by using 50 mg/L Hygromycin B and 200 mg/L antibacterial Cefotaxime. The transgenic *N. tabacum* were cultured in MS medium with 1.25 mM Pi concentration (HP) and 0.0625 mM Pi concentration (LP) for 7 days.

### Determination of Available Pi Content

The content of available Pi in plants was determined by the ammonium molybdate method (Nanamori et al., 2004). To determine the Pi concentration of the transgenic *N. tabacum*, the transgenic *N. tabacum* was ground with liquid nitrogen and 10% (w/v) perchloric acid (PCA). The supernatant was centrifuged after 10 times dilution with 5% (w/v) PCA. The working solution [sulfuric acid-ammonium molybdate (solution A) and ascorbic acid solution (solution B) were mixed in proportion (6:1)] extracts the available Pi from the supernatant. The absorbance was measured at 820 nm by a UV spectrophotometer.

### Quantitative Real-Time PCR Analysis

The RNA was extracted from the Liquid nitrogen quick-frozen plant tissues using a Plant Total RNA Isolation Kit (Sangon Biotech, Shanghai, China). A One Step RT-qPCR Kit (BBI Life Science, Shanghai, China) was used to obtain cDNA. 2× TaqMan Fast qPCR Master Mix (BBI Life Science, China) was used to execute qRT-PCR. Reaction conditions were performed according to Liu’s method (Liu L. et al., 2021). The qRT-PCR primers were designed using NCBI PRIMER-BLAST^8^ (Supplementary Table 3). Each experiment was set up with three biological replicates, and the results were calculated using 2^–ΔΔ^*^CT^* method.

### The Temporal Expression Patterns Analysis of *DoSPXs*

The transcriptome data of 8 *D. officinale* tissues (root, stem, leaf, flower buds, column, lip, and sepal) were downloaded from the NCBI SRA database (PRJNA348403). Trimmomatic (Bolger et al., 2014) is used to filter and trim data. A retrieval file of *D. officinale* genome was established by HISAT2 (Pertea et al., 2016), and high-quality reads were compared to *D. officinale* genome. Samtools (Li et al., 2009) was used for sorting and format conversion to obtain BAN format files. Finally, StringTie (Pertea et al., 2016) was assembled for sequence, and transcript abundance was estimated. TBOOLS (Chen et al., 2020) is used for the visualization of results.

## Results

### Identification of *SPX* Family Genes in *Dendrobium officinale*

By blasting the published genome sequences of *D. officinale* using the conserved SPX sequences reported in *O. sativa* and *A. thaliana*, the possible DoSPX sequences were obtained, and the redundancy of the sequences with high similarity was removed, then verified these sequences with SMART and PFAM, the result was shown in Table 1. Seven possible DoSPX sequences were obtained, all of which contain SPX conserved domains. Combining with the naming method of *A. thaliana*, two of them contain EXS domains named DoSPX-EXS1 and DoSPX-EXS2. One sequence contains the MFS sequence, named DoSPX-MFS. A sequence containing the RING field, called DoSPX-RING. The three sequences containing only the SPX domain were named DoSPX1, DoSPX3, and DoSPX4. The amino acid sequence analysis showed that the size of all proteins was 2.75–10.12 kDa and the isoelectric point was 5.08–9.22. The average hydrophilic coefficient (GRAVY < 0) shows that the other six are hydrophilic proteins except for DoSPX-MFS.

**TABLE 1:** Analysis of amino acid sequence encoded by DoSPXs.

Gene ID	Gene name	Length (bp)	Molecular weight (Da)	Theoretical Pi	Grand average of hydropathicity	Stable yes/no
MA16_Dca006348	DoSPX1	309	35431.5	5.08	−0.519	no
MA16_Dca004880	DoSPX3	241	27578.0	6.48	−0.341	no
MA16_Dca005615	DoSPX4	280	32060.3	5.48	−0.568	no
MA16_Dca009356	DoSPX-EXS1	574	65766.8	9.22	−0.283	yes
MA16_Dca005298	DoSPX-EXS2	871	101250.0	9.12	−0.249	yes
MA16_Dca004391	DoSPX-MFS	692	76992.1	5.87	0.226	yes
MA16_Dca006440	DoSPX-RING	286	32299.0	6.64	−0.199	no

### System Evolution Analysis of DoSPXs

**FIGURE 1:**
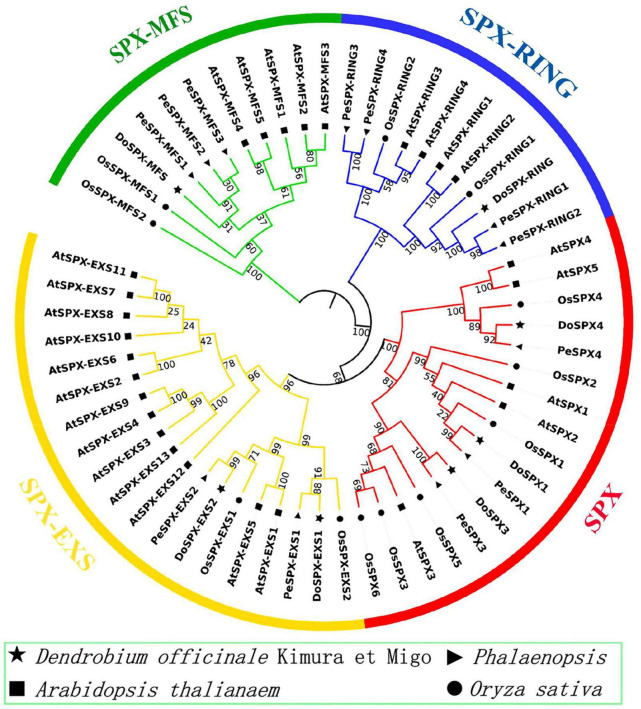
Phylogenetic analysis of SPX proteins in *Dendrobium officinale*, *Phalaenopsis equestris*, *Oryza sativa*, and *Arabidopsis thaliana*. The neighbor-joining (NJ) tree was created with 9 *D. officinale*, 12 *P. equestris*, 12 *O. sativa*, and 27 *A. thaliana* SPX protein sequences.

Through CLUSTERW alignment, the phylogenetic trees of *D. officinale*, *P. equestris*, *O. sativa*, and *A. thaliana* were established by the NJ method of MEGA7.0. The result was shown in Figure 1. It was found that all sequences were divided into four subclasses. The sequences of each subclass of SPX in *D. officinale* were well classified from those in *O. sativa* and *A. thaliana*. DoSPX1 is clustered with AtSPX1, 2, and OsSPX1; DoSPX3 were clustered with OsSPX3, 5, 6, and AtSPX3; while DoSPX4 is clustered with AtSPX4, OsSPX4. DoSPX-EXS1, DoSPX-EXS2, DoSPX-MFS, and DoSPX-RING are well clustered with the three subfamilies reported in *O. sativa* and *A. thaliana*.

### Analysis of *DoSPXs Cis*-Acting Elements

**FIGURE 2:**
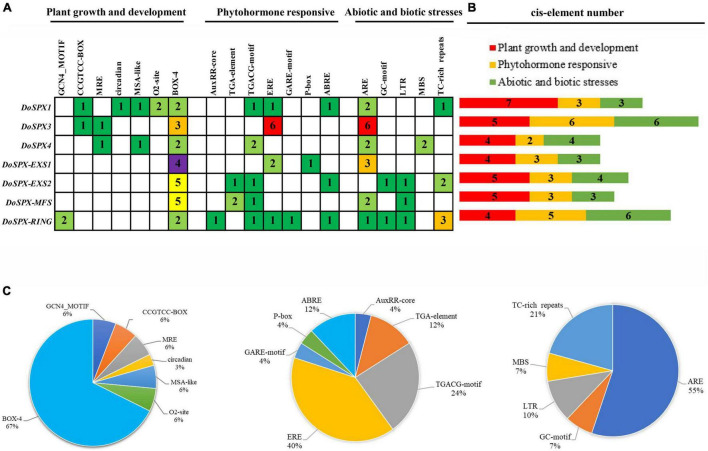
Analysis of the *cis*-acting elements in *DoSPXs* of *D. officinale*. **(A)** The number in each cell represents the number of *cis*-acting elements in different *SPX* genes. **(B)** Each bar diagram represents a different type of *cis*-acting element. **(C)** The pie chart represents the proportion of promoters in other groups.

By analyzing the upstream promoter sequence of *DoSPXs*, many *cis*-acting elements necessary for plant growth, development, and stress response were excavated. The result was shown in Figure 2. In these elements, MSA like, CAT-box and BOX-4 related to plant growth and development; ERE and TGACG-motif were involved in response to hormones; TC-rich repeat were involved in response to abiotic stress. These results suggested that *DoSPXs* may participate in the growth, development, and stress response of *D. officinale*.

### Expression Pattern Analysis of DoSPXs

**FIGURE 3:**
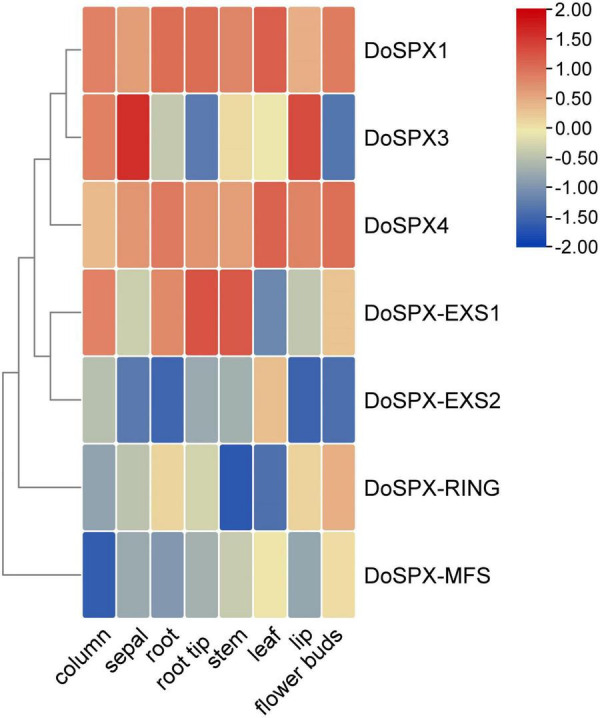
Expression profiles of *DoSPX* genes in 8 tissues of *D. officinale*. Blue to red indicates low to high expression levels of the gene.

In order to understand the expression patterns of the *DoSPXs* in *D. officinale*, the expression level of the *DoSPXs* in different tissues was analyzed. The results were presented in heat map form in Figure 3. *DoSPX1* and *DoSPX4* showed similar expression patterns in various tissues and had high expression levels in *D. officinale*. *DoSPX3* had high expression level in sepals, showed that *DoSPX*3 played an important role in the sepals. *DoSPX*-*EXS1* had high expression level in the root tips and stem. *DoSPX*-*EXS1*, *DoSPX*-*RING*, and *DoSPX*-*MFS* had low expression levels in all eight tissues.

**FIGURE 4:**
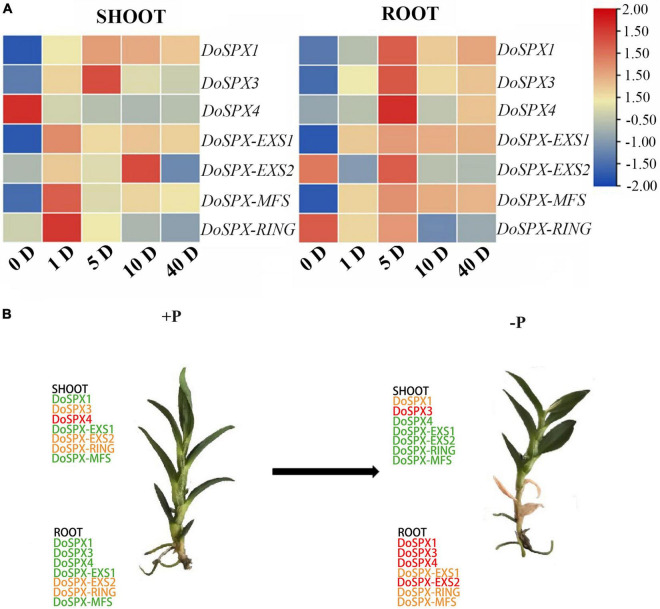
**(A)** Temporal and spatial expression patterns of *PSI* gene in shoot and roots under −P stress in *D. officinale*. The actin was used as an internal reference gene to regulate gene expression **(B)** *SPX* genes expression in shoot and root of *D. officinale*. Treated with −Pi for 5 day. Green, yellow, and red indicate low, medium, and high expression.

In order to understand the response of *DoSPXs* to −Pi stress, *D. officinale* was taken at 1, 5, 10, and 40 days after the −Pi treatment and determined the expression level of *DoSPXs*. The results were shown in Figure 4. Under the −P, the transcript abundance of most *PSI* genes increased. However, each gene showed its unique expression pattern at 0 day of the −P treatment. In *D. officinale* buds, the expression levels of *DoSPX*-*EXS1*, *DoSPX-MFS*, and *DoSPX-RING* reached the highest at 1 day after the −P treatment, *DoSPX1* and *DoSPX3* reached the highest at 5 day after the −P treatment; *DoSPX*-*EXS2* peaked at day 10. In roots, *DoSPX1*-*DoSPX4*, *DoSPX*-*EXS1*, *DoSPX-EXS2*, and *DoSPX*-*MFS* reached the highest expression level at day 5 after the −P treatment; their expression level increased with the increase of −Pi treatment time. These results suggested that *DoSPXs* mostly reached a high expression level in the early stage of −P stress, while *DoSPX4* showed different changes from other genes in the buds under the −P stress. The expression of *DoSPX4* reached its highest level at 0 day of −P treatment, and the expression down-regulated with −Pi treatment time.

### Subcellular Localization of DoSPX4

**FIGURE 5:**
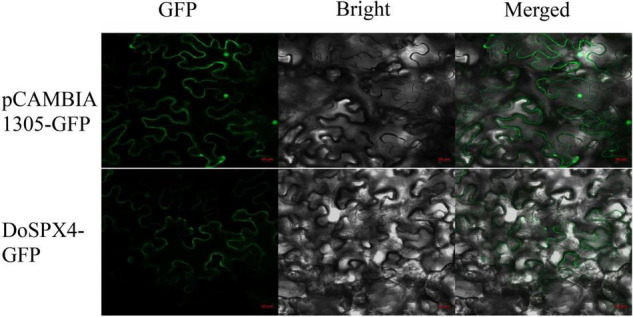
Subcellular localization of DoSPX4 protein in *Nicotiana tabacum* epidermal cells. The results were observed by confocal laser microscopy. The pictures show a green fluorescent field, a bright field and an overlay of two fields (merge). The numerical reading of the red ruler is 20 μm.

The CDS region without the stop codon of the *DoSPX4* gene was cloned into the pCAMBIA1305.1-GFP vector, and the subcellular localization of DoSPX4 in epidermal cells of *N. tabacum* was observed by a laser confocal microscope. The results showed that DoSPX4-GFP recombinant protein had a fluorescence signal on the cell membrane, while GFP was distributed in the whole cell (Figure 5).

### DoSPX4 Interact With DoPHR2 by Y2H

**FIGURE 6:**
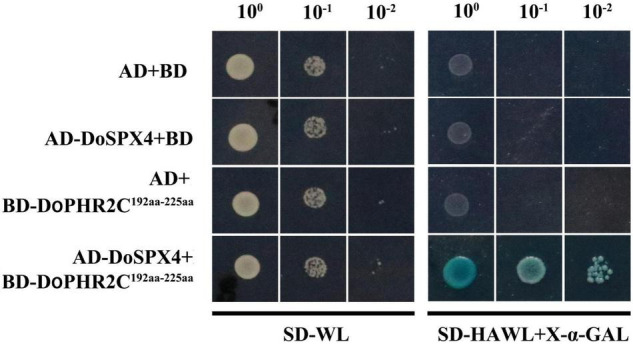
The DoSPX4 interacts with DoPHR2. The pGADT7 and pGBKT7 were used as the negative control. SD-WL indicates the SD medium without Leu and Trp, and SD-HAWL means the SD medium without Ade, His, Leu, and Trp.

To identify the interaction between DoSPX4 and DoPHR2, the CDS of DoSPX4 was cloned into pGADT7, and the segment (C192aa-225aa) of DoPHR2 without transcriptional activation domain was cloned into the pGBKT7 vector. AD-DoSPX4 and BD-DoPHR2^192aa–225aa^ were transformed into yeast strain AH109. The yeast strain could grow normally on SD/-Trp-Leu-His-Ade medium, indicating that DoSPX4 was tender enough to interact with DoPHR2^192aa–225aa^ (Figure 6).

### Overexpression *DoSPX4* in *Nicotiana tabacum*

**FIGURE 7:**
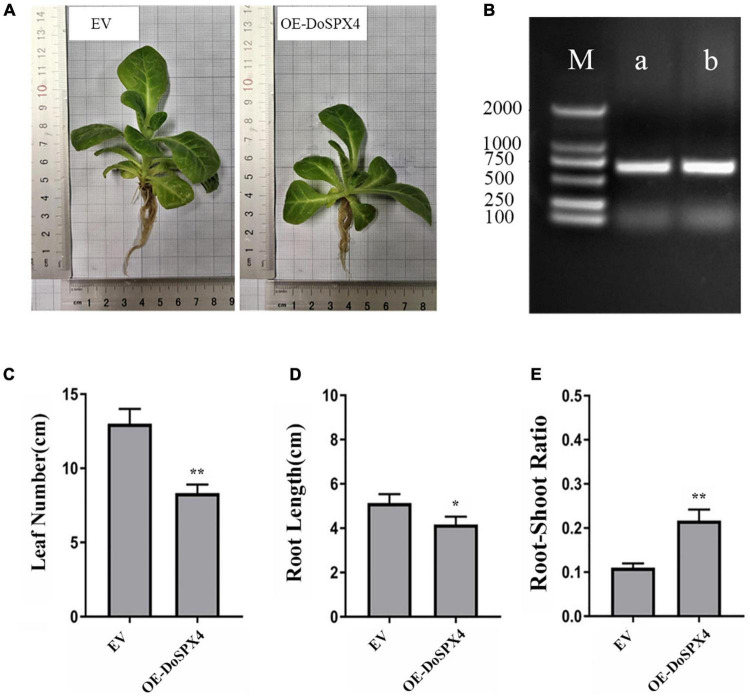
Transgene analysis. **(A)** Phenotypic difference of OE-*DoSPX4* transgenic *N. tabacum*. **(B)** Identification of positive transgenic *N. tabacum* lines by PCR (M represent DL2000 DNA marker, a represent EMPTY VECTOR, b represent OE-*DoSPX4*). **(C)** The leaf number of OE-*DoSPX4* transgenic *N. tabacum.* **(D)** The root length of OE-*DoSPX4* transgenic *N. tabacum.* **(E)** The root–shoot ratio of OE-*DoSPX4* transgenic *N. tabacum.* Each sample included at least three replicates, and the values are the means ± *SDs*. *Indicates that the difference is significant, **Indicates that the difference is very significant.

To analyze the function of *DoSPX4* in plants, we obtained transgenic *N. tabacum* overexpression *DoSPX4*. As shown in Figure 7, through −Pi treatment, the length of leaves and root in OE-*DoSPX*4 *N. tabacum* plants decreased significantly, and the root–shoot ratio was 1.97 times than that of the empty vector (EV) group.

To understand the function of *DoSPXs* on Pi absorption and utilization, the available Pi contents of transgenic *N. tabacum* under + P and −P culture were measured (Figure 7). Under the −P stress, P content in the aboveground part of OE-*DoSPX4 N. tabacum* was significantly higher than that in the EV group. These results indicated that the *DoSPX4* overexpression promoted the Pi accumulation in the shoot.

**FIGURE 8:**
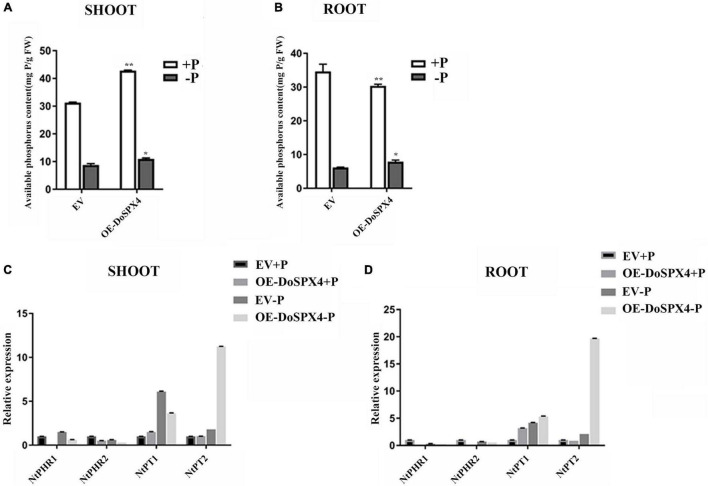
Determination of indices in transgenic *N. tabacum*. **(A)** The available Pi concentration in shoot in OE-*DoSPX4 N. tabacum*. **(B)** The available Pi concentration in root in OE-*DoSPX4 N. tabacum*. **(C)** The expression analysis of *NtPHR1/2* and *NtPT1/2* in shoot in OE-*DoSPX4 N. tabacum*. **(D)** The expression analysis of *NtPHR1/2* and *NtPT1/2* in root in OE-*DoSPX4 N. tabacum*. Each sample included at least three replicates, and the values are the means ± SDs. *Indicates that the difference is significant, **Indicates that the difference is very significant.

The expression levels of Pi response genes (*NtPHRs* and *NtPTs*) in OE-*DoSPX4 N. tabacum* were also detected (Figure 8). Under + P, the expression levels of *NtPHR1* and *NtPHR2* were significantly lower in OE-*DoSPX4 N. tabacum* than those in the control group. Under the −P condition, the expression levels of *NtPHR1* and *NtPHR2* were significantly up-regulated except the *NtPHR2* in the aboveground of OE-*DoSPX4 N. tabacum*. *NtPT1* and *NtPT2* were basically induced in OE-*DoSPX4 N. tabacum*, especially under the −Pi stress. These results suggested that *DoSPX4* may play a negative regulatory role in the expression of *NtPHR1* and *NtPHR2*, and then affect the process of Pi transport from *N. tabacum* roots to shoot.

## Discussion

The organic Pi that plants can absorb in the environment cannot satisfy the need of plants (Grennan, 2008). Medicinal plants have also evolved complex mechanisms to adapt to the −Pi responses (Liu L. et al., 2016, 2018; Wang et al., 2020). The proteins, containing the SPX domain, participate in the molecular regulatory network of plant response to Pi stress (Liu N. et al., 2018). Through the genome analysis of *D. officinale*, we obtained seven DoSPX proteins. DoSPX proteins have strong homology in *P. equestris* and *D. officinale*, suggesting that the regulatory network of plants responding to −Pi stress may be conserved. Through phylogenetic analysis and conservative motif analysis, we found that seven DoSPXs belong to 4 subfamilies (SPX, SPX-MFS, SPX-EXS, and SPX-RING) and have homology with *O. sativa* and *A. thaliana*.

The *cis*-acting elements of *DoSPXs* promoter region include MSA-like elements involved in cell cycle regulation and CAT-box elements involved in meristem development, which indicates that DoSPXs may be involved in the growth of *D. officinale*. In addition, the promoter region contains ERF, TGACG-motif, TC-rich, and ARE elements, which can respond to ethylene, plant hormones, plant defense and stress (Feng et al., 2020; Huo et al., 2021; Zhang et al., 2022). These results indicated that DoSPXs may involve in abiotic stress such as invasion and drought.

Tissue special expression showed that *DoSPXs* had different expression patterns, which indicates that *DoSPXs* may play different functions. The expression level of *DoSPX1* and *DoSPX3* was induced in the roots and shoot of *D. officinale* under −P and reached the peak at day 5, which indicated that *DoSPX1* and *DoSPX3* may participate in the early response of *D. officinale* to −P, this was similar to the expression pattern of *AtSPX1/2/3* (Duan et al., 2008; Puga et al., 2014) and O*sSPX1/2/3/5/6* (Wang et al., 2009; Shi et al., 2014).

Under the −P, DoSPX4 had a different expression pattern compared to *DoSPX1* and *DoSPX3*. According to homology comparison analysis, DoSPX4 had high homology with OsSPX4 (Lv et al., 2014) and AtSPX4 (Duan et al., 2008). In *A. thaliana*, the localization of AtSPX4 is different from AtSPX1 and AtSPX3, which shows the uniqueness of the function of AtSPX4 in *A. thaliana* Pi response. The subcellular localization analysis indicates that DoSPX4 is located on the membrane, which is consistent with AtSPX4 and OsSPX4. These results imply that DoSPX4 may have similar functions to AtSPX4 and OsSPX4, but this still needs a lot of experimental to prove.

It has been reported that SPX can interact with MYB-CC transcription factor PHR and then affect the downstream PSI genes (Lv et al., 2014). InsPs can promote the interaction between SPX and the MYB-CC domain of PHR (Duan et al., 2008). The C-terminal of DoPHR2 contains the MYB-CC domain. At Yeast two-hybrid test showed that DoSPX4 interacted with the C-terminal of DoPHR2. The results indicated that DoSPX4 worked by DoPHR2 in −P. However, the recognition of SPX protein conserved domain by InsPs and the regulation of DoPHR2 at the protein level need an in-depth study.

**FIGURE 9:**
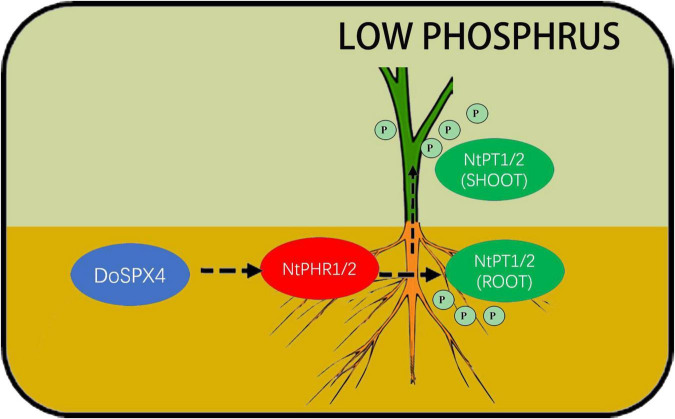
Possible DoSPX4 impact mechanism. *DoSPX4* promoted the expression of *NtPHR1/2* in roots, induced the expression of *NtPT1/2* and high-affinity phosphate transporter in roots and shoot, and promoted the transportation of Pi from roots to stems in transgenic *N. tabacum* in low phosphorus.

The *DoSPX4* was overexpressed in *N. tabacum*, which can observe the changes in the root system, Pi content, and Pi transporter expression levels. In OE-*DoSPX4 N. tabacum*, the root–shoot ratio increased, which was conducive to the absorption of Pi from the environment. the qRT-PCR analysis found that the expression of *NtPHR1/2* and *NtPT1/2* in OE-*DoSPX4* transgenic *N. tabacum* shoot and root increased. The effective Pi content decreased in the root, while the effective Pi content increased in the aboveground part. It is speculated that *DoSPX4* is involved in the induction of *NtPT1/2* by NtPHR1/2. *NtPT1/2* is a high-affinity phosphate transporter responsible for the long-distance transport of Pi in plants. The high expression of *NtPT1/2* promotes the transport of Pi from root to aboveground part *in vivo*, which increases the Pi content of aboveground parts. The absorption and utilization efficiency of Pi in *N. tabacum* was improved (Figure 9).

## Conclusion

In this study, seven SPX family proteins were identified from *D. officinale*. The qRT-PCR analysis showed the SPX family had different expression patterns. The DoSPX4 was located in the cell membrane and had the ability to interact with DoPHR2. The heterologous expression of *N. tabacum* showed that *DoSPX4* could activate *NtPHR1/2*, increase the expression of *NtPT1/2* in roots and stems, and promote the transport of Pi from roots to shoot. These results provide an experimental basis for further study on the adaptation mechanism of *D. officinale* to low phosphorus.

## Data Availability Statement

The datasets presented in this study can be found in online repositories. The names of the repository/repositories and accession number(s) can be found in the article/Supplementary Material.

## Author Contributions

HF organized and designed the experiment. LL and HX conducted the experiment and completed the manuscript writing. JS and HS analyzed the data and put forward valuable suggestions to XS and LT in the process of manuscript modification. All authors read and approved the manuscript.

## Conflict of Interest

The authors declare that the research was conducted in the absence of any commercial or financial relationships that could be construed as a potential conflict of interest.

## Publisher’s Note

All claims expressed in this article are solely those of the authors and do not necessarily represent those of their affiliated organizations, or those of the publisher, the editors and the reviewers. Any product that may be evaluated in this article, or claim that may be made by its manufacturer, is not guaranteed or endorsed by the publisher.

## Footnotes

1
http://weizhong-lab.ucsd.edu/cdhit_suite/cgi-bin/index.cgi

2
http://smart.embl-heidelberg.de/

3
http://pfam.xfam.org/

4
http://web.expasy.org/protparam/

5
https://itol.embl.de/

6
http://bioinformatics.psb.ugent.be/webtools/plantcare/html/

7
https://www.dna.affrc.go.jp/PLACE/?action=newplace

8
https://www.ncbi.nlm.nih.gov/tools/primer-blast/

## References

[B1] Bhatt P. Rene E. R. Huang Y. H. Lin Z. Q. Pang S. M. Zhang W. P. (2021). Systems biology analysis of pyrethroid biodegradation in bacteria and its effect on the cellular environment of pests and humans. *J. Environ. Chem. Eng*. 9:106582. 10.1016/j.jece.2021.106582

[B2] Bolger A. M. Lohse M. Usadel B. (2014). Trimmomatic: a flexible trimmer for illumina sequence data. *Bioinformatics* 30 2114–2120. 10.1093/bioinformatics/btu170 24695404 PMC4103590

[B3] Chen C. J. Chen H. Zhang Y. Thomas H. R. Frank M. H. He Y. H. (2020). TBtools: an integrative toolkit developed for interactive analyses of big biological data. *Mol. Plant*. 13 1194–1202. 10.1016/j.molp.2020.06.009 32585190

[B4] Chen L. Liao H. (2017). Engineering crop nutrient efficiency for sustainable agriculture. *J. Integr. Plant Biol*. 59 710–735. 10.1111/jipb.12559 28600834

[B5] Chen Y. F. Li L. Q. Xu Q. Kong Y. H. Wang H. Wu W. H. (2009). The WRKY6 transcription factor modulates *PHOSPHATE1* expression in response to low Pi stress in *Arabidopsis*. *Plant Cell* 21 3554–3566. 10.1105/tpc.108.064980 19934380 PMC2798333

[B6] Chiou T. J. Lin S. I. (2011). Signaling network in sensing phosphate availability in plants. *Annu. Rev. Plant Biol*. 62 185–206. 10.1146/annurev-arplant-042110-103849 21370979

[B7] Duan K. Yi K. K. Dang L. Huang H. J. Wu W. Wu P. (2008). Characterization of a sub-family of Arabidopsis genes with the SPX domain reveals their diverse functions in plant tolerance to phosphorus starvation. *Plant J*. 54 965–975. 10.1111/j.1365-313X.2008.03460.x 18315545

[B8] Feng K. Hou X. L. Xing G. M. Liu J. X. Duan A. Q. Xu Z. S. (2020). Advances in AP2/ERF super-family transcription factors in plant. *Crit. Rev. Biotechnol*. 40 750–776. 10.1080/07388551.2020.1768509 32522044

[B9] Fu L. M. Niu B. F. Zhu Z. W. Wu S. T. Li W. Z. (2012). CD-HIT: accelerated for clustering the next-generation sequencing data. *Bioinformatics* 28 3150–3152. 10.1093/bioinformatics/bts565 23060610 PMC3516142

[B10] Gasteiger E. Gattiker A. Hoogland C. Ivanyi I. Appel R. D. Bairoch A. (2003). ExPASy: the proteomics server for in-depth protein knowledge and analysis. *Nucleic Acids Res.* 31 3784–3788. 10.1093/nar/gkg563 12824418 PMC168970

[B11] Grennan A. K. (2008). Phosphate accumulation in plants: signaling. *Plant Physiol*. 148 3–5. 10.1104/pp.104.900269 18772350 PMC2528084

[B12] Higo K. Ugawa Y. Iwamoto M. Korenaga T. (1999). Plant cis-acting regulatory DNA elements (PLACE) database: 1999. *Nucleic Acids Res*. 27 297–300. 10.1093/nar/27.1.297 9847208 PMC148163

[B13] Huo Y. B. Zhang B. Chen L. Zhu C. Zhang X. Zhu C. (2021). Isolation and functional characterization of the promoters of miltiradiene synthase genes, *TwTPS27a* and *TwTPS27b*, and interaction analysis with the transcription factor TwTGA1 from *Tripterygium wilfordii*. *Plants (Basel)* 10:418. 10.3390/plants10020418 33672407 PMC7926782

[B14] Lescot M. Déhais P. Thijs G. Zhang J. Zhang X. Zhu C. S. (2002). PlantCARE, a database of plant cis-acting regulatory elements and a portal to tools for in silico analysis of promoter sequences. *Nucleic Acids Res*. 30 325–327. 10.1093/nar/30.1.325 11752327 PMC99092

[B15] Letunic I. Bork P. (2021). Interactive Tree Of Life (iTOL) v5: an online tool for phylogenetic tree display and annotation. *Nucleic Acids Res*. 49 W293–W296. 10.1093/nar/gkab301 33885785 PMC8265157

[B16] Letunic I. Khedkar S. Bork P. (2021). SMART: recent updates, new developments and status in 2020. *Nucleic Acids Res*. 49 D458–D460. 10.1093/nar/gkaa937 33104802 PMC7778883

[B17] Li H. Handsaker B. Wysoker A. Fennell T. Ruan J. Homer N. (2009). 1000 genome project data processing subgroup The sequence alignment/map (SAM) format and SAMtools. *Bioinformatics* 25 2078–2079. 10.1093/bioinformatics/btp352 19505943 PMC2723002

[B18] Lin W. Y. Huang T. K. Chiou T. J. (2013). Nitrogen limitation adaptation, a target of microRNA827, mediates degradation of plasma membrane-localized phosphate transporters to maintain phosphate homeostasis in *Arabidopsis*. *Plant Cell* 25 4061–4074. 10.1105/tpc.113.116012 24122828 PMC3877804

[B19] Liu J. Fu S. Yang L. Luan M. Zhao F. Luan S. (2016). Vacuolar SPX-MFS transporters are essential for phosphate adaptation in plants. *Plant Signal. Behav*. 11:e1213474. 10.1038/ncomms11095 27467463 PMC5022419

[B20] Liu L. Xiang H. X. Shen H. M. Dong Y. X. Sun X. Cai Y. P. (2021). Effects of low phosphorus stress on the main active ingredients and antioxidant activities of *Dendrobium officinale*. *Ind. Crop Prod*. 173:114095. 10.1016/j.indcrop.2021.114095

[B21] Liu L. Yang D. F. Liang T. Y. Zhang H. H. Liang Z. S. (2016). Phosphate starvation promoted the accumulation of phenolic acids by inducing the key enzyme genes in *Salvia miltiorrhiza* hairy roots. *Plant Cell Rep*. 35 1933–1942. 10.1007/s00299-016-2007-x 27271760

[B22] Liu L. Yang D. F. Xing B. C. Zhang H. Liang Z. (2018). *Salvia castanea* hairy roots are more tolerant to phosphate deficiency than *Salvia miltiorrhiza* hairy roots based on the secondary metabolism and antioxidant defenses. *Molecules* 23:1132. 10.3390/molecules23051132 29747474 PMC6099837

[B23] Liu N. Shang W. Li C. Jia L. H. Wang X. Xing G. (2018). Evolution of the *SPX* gene family in plants and its role in the response mechanism to phosphorus stress. *Open Biol*. 8:170231. 10.1098/rsob.170231 29298909 PMC5795055

[B24] Lv Q. Zhong Y. Wang Y. Zhang L. Shi J. Wu Z. (2014). SPX4 negatively regulates phosphate signaling and homeostasis through its interaction with PHR2 in rice. *Plant Cell* 26 1586–1597. 10.1105/tpc.114.123208 24692424 PMC4036573

[B25] Mistry J. Chuguransky S. Williams L. Qureshi M. Salazar G. A. Sonnhammer E. L. L. (2021). Pfam: the protein families database in 2021. *Nucleic Acids Res*. 49 D412–D419. 10.1093/nar/gkaa913 33125078 PMC7779014

[B26] Nanamori M. Shinano T. Wasaki J. Yamamura T. Rao I. M. Osaki M. (2004). Low phosphorus tolerance mechanisms: phosphorus recycling and photosynthate partitioning in the tropical forage grass, *Brachiaria* hybrid cultivar Mulato compared with rice. *Plant Cell Physiol*. 45 460–469. 10.1093/pcp/pch056 15111721

[B27] Osorio M. B. Ng S. Berkowitz O. De Clercq I. Mao C. Z. Shou H. X. (2019). SPX_4_ acts on phr_1_-dependent and -independent regulation of shoot phosphorus status in arabidopsis. *Plant Physiol.* 1, 332–352. 10.1104/pp.18.00594 31262954 PMC6716250

[B28] Pertea M. Kim D. Pertea G. M. Leek J. T. Salzberg S. L. (2016). Transcript-level expression analysis of RNA-seq experiments with HISAT, StringTie and Ballgown. *Nat. Protoc.* 11 1650–1667. 10.1038/nprot.2016.095 27560171 PMC5032908

[B29] Puga M. I. Mateos I. Charukesi R. Wang Z. Franco-Zorrilla J. M. de Lorenzo L. (2014). SPX1 is a phosphate-dependent inhibitor of phosphate starvation response 1 in *Arabidopsis*. *Proc. Natl. Acad. Sci. U.S.A*. 111 14947–14952. 10.1073/pnas.1404654111 25271326 PMC4205628

[B30] Secco D. Wang C. Arpat B. A. Wang Z. Y. Poirier Y. Tyerman S. D. (2012). The emerging importance of the SPX domain-containing proteins in phosphate homeostasis. *New Phytol.* 4, 842–851. 10.1111/j.1469-8137.2011.04002.x 22403821

[B31] Shi J. Hu H. Zhang K. Zhang W. Yu Y. M. Wu Z. (2014). The paralogous *SPX3* and *SPX5* genes redundantly modulate Pi homeostasis in rice. *J. Exp. Bot.* 65 859–870. 10.1093/jxb/ert424 24368504 PMC3924727

[B32] Su T. Xu Q. Zhang F. C. Chen Y. Li L. Q. Wu W. H. (2015). WRKY42 modulates phosphate homeostasis through regulating phosphate translocation and acquisition in *Arabidopsis*. *Plant Physiol*. 167 1579–1591. 10.1104/pp.114.253799 25733771 PMC4378159

[B33] Thompson J. D. Higgins D. G. Gibson T. J. (1994). CLUSTAL W: improving the sensitivity of progressive multiple sequence alignment through sequence weighting, position-specific gap penalties and weight matrix choice. *Nucleic Acids Res*. 22 4673–4680. 10.1093/nar/22.22.4673 7984417 PMC308517

[B34] Topping J. F. (1988). Tobacco transformation. *Methods Mol. Biol*. 81 362–372. 10.1385/0-89603-385-6:365 9760526

[B35] Wang B. Zhang T. X. Du H. W. Zhao Q. Meng X. C. (2020). Effect of peg on secondary metabolites in suspension cells of scutellaria baicalensis georgi. *Acta Med. Mediterr*. 36 2307–2312. 10.19193/0393-6384_2020_4_359

[B36] Wang C. Yue W. Ying Y. Wang S. Secco D. Liu Y. (2015). Rice SPX-major facility superfamily3, a vacuolar phosphate efflux transporter, is involved in maintaining phosphate homeostasis in rice. *Plant Physiol*. 169 2822–2831. 10.1104/pp.15.01005 26424157 PMC4677894

[B37] Wang Y. Ribot C. Rezzonico E. Poirier Y. (2004). Structure and expression profile of the Arabidopsis *PHO1* gene family indicates a broad role in inorganic phosphate homeostasis. *Plant Physiol*. 135 400–411. 10.1104/pp.103.037945 15122012 PMC429393

[B38] Wang Z. Y. Hu H. Huang H. J. Duan K. Wu Z. C. Wu P. (2009). Regulation of OsSPX1 and OsSPX3 on expression of *OsSPX* domain genes and pistarvation signaling in rice. *J. Integr. Plant Biol.* 51, 663–674. 10.1111/j.1744-7909.2009.00834.x 19566645

[B39] Wang Z. Y. Ruan W. Y. Shi J. Zhang L. Xiang D. Yang C. (2014). Rice SPX1 and SPX2 inhibit phosphate starvation responses through interacting with PHR2 in a phosphate-dependent manner. *Proc. Natl. Acad. Sci. U.S.A*. 111 14953–14958. 10.1073/pnas.1404680111 25271318 PMC4205599

[B40] Yang J. Wang L. Mao C. Z. Lin H. H. (2018). Characterization of the rice NLA family reveals a key role for OsNLA1 in phosphate homeostasis. *Rice* 10:52. 10.1186/s12284-017-0193-y 29282559 PMC5745205

[B41] Yue W. H. Ying Y. H. Wang C. Zhao Y. Dong C. H. Whelan J. (2017). OsNLA1, a RING-type ubiquitin ligase, maintains phosphate homeostasis in *Oryza sativa via* degradation of phosphate transporters. *Plant J*. 90 1040–1051. 10.1111/tpj.13516 28229491

[B42] Zhang B. Song Y. F. Zhang X. D. Wang Q. N. Li X. Q. He C. Z. (2022). Identification and expression assay of calcium-dependent protein kinase family genes in Hevea brasiliensis and determination of HbCDPK5 functions in disease resistance. *Tree Physiol*. 42 1070–1083. 10.1093/treephys/tpab156 35022787

[B43] Zhang J. Y. Zhou X. Xu Y. Yao M. L. Xie F. B. Gai J. Y. (2016). Soybean SPX1 is an important component of the response to phosphate deficiency for Pi homeostasis. *Plant Sci*. 248 82–91. 10.1016/j.plantsci.2016.04.010 27181950

[B44] Zhou J. Hu Q. L. Xiao X. L. Yao D. Q. Ge S. H. Ye J. (2021). Mechanism of phosphate sensing and signaling revealed by rice SPX1-PHR2 complex structure. *Nat. Commun*. 12:7040. 10.1038/s41467-021-27391-5 34857773 PMC8639918

